# Plasma-Treated Water (PTW) Delaying the Lag Phase of Food-Related Pathogens

**DOI:** 10.3390/microorganisms14081832

**Published:** 2026-08-19

**Authors:** Laura Hartwig, Samantha Nestel, Mareike Meister, Jörg Ehlbeck, Robert Wagner, Uta Schnabel

**Affiliations:** Leibniz Institute for Plasma Science and Technology, Felix-Hausdorff-Strasse 2, 17489 Greifswald, Germany; laura.hartwig@stud.uni-greifswald.de (L.H.); samantha.nestel@inp-greifswald.de (S.N.); mareike.meister@inp-greifswald.de (M.M.); ehlbeck@inp-greifswald.de (J.E.); robert.wagner@inp-greifswald.de (R.W.)

**Keywords:** foodborne pathogens, biofilms, growth curves, reactive oxygen and nitrogen species (RONS), optical density, colony-forming units, *Escherichia coli*, *Bacillus cereus*, *Listeria monocytogenes*, *Staphylococcus aureus*

## Abstract

Foodborne pathogens such as *E. coli*, *B. cereus*, *L. monocytogenes*, and *S. aureus* cause many illnesses in Europe each year due to traits like biofilm formation, spore production, and high resistance to harsh conditions. This study investigated plasma-treated water (PTW) as an eco-friendly antimicrobial approach and examined its effect on bacterial growth curves to improve food safety. PTW was generated using a microwave plasma source, then applied to bacterial cultures that were monitored by optical density and colony counts. The bacterial growth was described by application of a logistic growth model to the optical density and colony count data. Results show that PTW mainly prolonged the lag phase of growth, with a stronger effect on Gram-positive bacteria than on Gram-negative bacteria. Although PTW initially reduced viable cell numbers, most bacteria eventually recovered and reached the stationary phase after a delay of about 2 to 11 h depending on the species. These effects may be due to cell damage, cell death, viable but nonculturable state induction, or stress responses such as spore formation. Further studies are needed to confirm how PTW performs in real food systems and biofilm settings.

## 1. Introduction

In 2023, there were 5691 foodborne outbreaks, events in which two or more people contract the same illness from the same food, reported by the European Food Safety Authority (EFSA) [1]. Of these outbreaks, approximately 2400 were caused by bacteria or bacterial toxins. Pathogens such as *Escherichia coli*, *Bacillus cereus*, *Listeria monocytogenes*, and *Staphylococcus aureus* have been listed as common causative agents in foodborne illness reports for decades [2,3,4]. These pathogens can lead to illnesses that result in severe symptoms, hospitalization, and even death. While these pathogens vary in size, shape, and other characteristics, they all possess unique properties that make them resilient to harsh conditions and, consequently, difficult to mitigate or kill.

*Escherichia coli* is a highly diverse, Gram-negative, non-spore forming, rod-shaped bacteria [5]. *E. coli* can easily form biofilms on living and non-living carriers, presenting a food processing safety concern, as biofilms are markedly more resistant to disinfectants compared to planktonic cells [6,7]. It is also a facultative anaerobe, with the ability to grow in the presence or absence of oxygen, giving it a selective advantage for infection [8]. *E. coli* can survive for long periods of time and is typically found in the digestive system of humans and animals. Therefore, foodborne outbreaks may indicate fecal contamination [3,9]. Most strains of *E. coli* are innocuous and play a critical role in the human microbiome; however, other strains are pathogenic and can produce harmful toxins [10]. Enterohemorrhagic *E. coli* (EHEC) and Shiga toxin-producing *E. coli* (STEC) are the most common pathotypes of *E. coli* that cause foodborne illness from undercooked meat, unpasteurized beverages, and raw fruits and vegetables [1,2]. *E. coli* infection can lead to symptoms such as bloody diarrhea, abdominal pain, and hemolytic uremia syndrome and was the reported causative agent of 953 cases of illness and 51 hospitalizations in Europe during 2023.

*Bacillus cereus* is a large, Gram-positive, and motile rod-shaped bacteria that has the ability to form spores [3]. These spores are hydrophobic, which enables them to attach well to different surfaces [11]. *B. cereus* is capable of forming biofilms by producing a matrix of exopolysaccharides, proteins, and extracellular DNA that increases its adhesion ability on various surfaces, including glass or polystyrene [12]. This increased adhesion from spore and biofilm formation make *B. cereus* resilient to removal from food products and equipment surfaces during sanitation procedures in food processing. Two types of toxins are produced by *B. cereus*: the emetic type, leading to rapid onset of nausea and vomiting, and the diarrheal type, causing abdominal pain and diarrhea [2]. Both the emetic and diarrheal type of syndromes are a result of *B. cereus* endospores surviving the cooking process of foods followed by the reactivation of the endospores into their vegetative state [3]. *B. cereus* toxins were responsible for 4665 reported foodborne illness cases and 101 hospitalizations in Europe during 2023 [1].

*Listeria monocytogenes* is a small, Gram-positive rod bacteria species [13]. It is non-spore forming; however, it is well known for its ability to survive for years under adverse conditions while remaining capable of contamination [14]. *L. monocytogenes* poses a food safety threat, as it can form resilient biofilms on various surfaces such as stainless steel, polystyrene, and glass within the food processing industry [15]. Along with biofilm formation, persistence of *L. monocytogenes* in the food environment is hypothesized to be a result of the inability to fully remove cells from hard-to-clean niches [16]. Unlike other foodborne pathogens, *L. monocytogenes* is capable of surviving and multiplying in the cold temperatures of refrigeration, presenting a risk to safety particularly in raw fruits and vegetables, deli meats, and ready-to-eat meals that do not undergo other processing, such as cooking, before being ingested [3,17]. This pathogen causes listeriosis, which is a severe illness with symptoms of muscle aches, nausea, and diarrhea. It is one of the leading causes of death from foodborne pathogens and is particularly dangerous for pregnant women, causing premature delivery or stillbirth, and for elderly adults, leading to meningitis. During 2023 in Europe, there were 133 reported cases of illness, 84 of which led to hospitalization and 11 deaths from *Listeria monocytogenes* [1].

*Staphylococcus aureus* is a Gram-positive, non-motile, and non-spore-forming bacteria [18]. It has a spherical (cocci) shape that is known for growing in “grapelike” clusters [19]. *S. aureus* is considered to be one of the most resistant non-spore-forming pathogens due to its ability to survive for extended periods in a dry state, such as on dry plastic or human skin, and its tolerance to high salt concentration [20,21,22]. It is also able to form biofilms that enhance its resistance to antibiotics, making treatment of infection difficult [23]. Staphylococci are ubiquitous and can be found in the air, water, meat and dairy products, and on many environmental surfaces [20]. Food contamination is common in meat, poultry, and dairy products and is typically caused by cross-contamination during processing with other food products or by poor hygiene during human food handling [3,20,24]. Although the bacteria itself can be destroyed by heat, *S. aureus* produces enterotoxins that are highly heat stable and can retain their biological activity that leads to illness. These enterotoxins can cause sudden nausea, vomiting, abdominal pain, and diarrhea upon infection [2]. *S. aureus* toxins caused 2268 reported cases of illness in Europe during 2023, with the highest number of hospitalizations from a bacterial toxin being 113 patients [1].

Nonthermal plasma presents innovative techniques for application within the food industry to increase food safety. Specifically, plasma-treated water (PTW) has gained interest for its ability to remove pathogens from the product directly or from surfaces within processing, while being an environmentally safe sanitation agent. In previous research studies, PTW has shown promising results as an effective antimicrobial agent against each of these target pathogens, among others [25,26,27]. Additionally, PTW treatment has proven to be effective at inactivating bacterial biofilms of each of these pathogens [28,29,30,31]. PTW contains reactive nitrogen and oxygen species (RONS) that disrupt cellular functions, such as causing membrane damage, reducing proliferation ability, and reducing metabolic activity of cells [30]. The RONS present in the PTW depend on the conditions during production, including the plasma source, working gas, and length of treatment time. The present study relied on a microwave plasma source known as the MidiPLexc (designed in-house by the Leibniz Institute of Plasma Science and Technology; Greifswald, Germany), operating with compressed atmospheric air. Under these conditions, the MidiPLexc induced the formation of RONS, mainly nitrite (NO_2_^−^), nitrate (NO_3_^−^), and hydrogen peroxide (H_2_O_2_) [32].

This study aims to analyze the effect of plasma-treated water on the growth curves of these harmful pathogens associated with foodborne illness. Specifically, we want to identify differences in stages of the growth curve after PTW treatment to understand the potential ways that treatment affects the growth and health of bacterial cells. The applied growth model is used to not only deepen hypotheses about possible effects of the PTW application from previous publications that only analyzed a single time point [33,34], but also to quantify differences between observed values over time compared to the previously obtained values. This knowledge can underpin further understanding about the optimization of PTW treatment applied in the food sector to prevent or mitigate bacteria contamination, particularly bacteria that have resistance mechanisms such as spore or biofilm formation. Analyzing the growth of these bacteria species under treatment conditions provides insight into potential methods to mitigate these pathogens and prevent foodborne illnesses caused by them.

## 2. Materials and Methods

### 2.1. Production of PTW Using Microwave Plasma Source

Plasma-treated water was generated using the MidiPLexc microwave-driven plasma source. The MidiPLexc is an adaptation of the MiniMIP plasma source but implements compressed atmospheric air as the working gas instead of an inert gas [30]. A 1 L glass bottle was securely fit to the plasma source to create an enclosed space where the plasma effluent treated the air inside the bottle. The plasma source ran with a forward power of 50 W and a reverse power up to 5% of the forward power and with a compressed air gas flow of 1.5 standard L/min (slm). The plasma source was allowed to run with an empty bottle for 15 min to ensure steady state. Then, another 1 L glass bottle with 100 mL of tap water at room temperature and with a magnetic stir bar was connected and treated for 90 min. The water was consistently stirred at 80 rpm during treatment. After treatment, the plasma source was switched off, and the bottle remained fastened to the source for another 10 min. Directly following treatment, the PTW was transferred to 50 mL tubes and stored at −20 °C. PTW was defrosted at 4 °C for 24 h prior to use on samples. Once thawed, the pH and conductivity were measured (probes from Mettler Toledo GmbH, Gießen, Germany) to ensure expected plasma treatment (pH ≤ 2 and conductivity ≈ 7000–8000 µS/cm). The experimentally used PTW had a pH of 1.6 ± 0.8 and conductivity of 8954 ± 636 µS/cm.

In the study by Yao et al. (2026), the long-living chemical components in plasma-processed air (PPA, the basis for PTW generation) generated were characterized using FTIR spectroscopy [32]. The main findings regarding the chemistry of PPA were as follows: (1) The dominant species were nitrogen monoxide (NO), nitrogen dioxide (NO_2_), and dinitrogen tetroxide (N_2_O). NO_2_ was the dominant compound, exceeding NO by more than one order of magnitude after 10 min of operation. (2) The concentrations of NO and NO_2_ increased over time, stabilizing after approximately 25 min. Under dry conditions, NO concentrations reached 576–723 ppm, while NO_2_ concentrations reached 5382 ppm. (3) Humidity was an influencing factor. When water vapor was present (100% relative humidity), additional bands characterizing nitric acid (HNO) were observed in the spectra, partially overlapping with N_2_O_4_. This indicates that under humid conditions, part of the NO_2_ gas was absorbed to produce HNO_3_. (4) No trace of ozone (O_3_) was detected by the FTIR spectrometer during the entire plasma operation, suggesting it was either consumed in reactions with NO to form NO_2_ or it reacted with the inner surface of the tubes before reaching the detector.

In the study by Nestel et al. (2025), the chemical composition of PTW is described and detailed [31]. The experimental setup for PTW generation and the chosen physical parameters (gas flow and treatment time of 90 min) were comparable. Therefore, it can be assumed that the concentrations of nitrite, nitrate and hydrogen peroxide were in the range of 112 mg/L, 2428 mg/L, and >196 µg/mL, respectively.

### 2.2. Overnight Culture and Solution Preparation

The ideal media were selected for the individual bacterial species. Tryptic Soy Broth (TSB) (Carl Roth GmbH & Co. KG, Karlsruhe, Germany) was used for culturing *E. coli* DSM 11250, *B. cereus* DSM 31, and *S. aureus* DSM 799, while Brain Heart Infusion (BHI) broth (Carl Roth GmbH & Co. KG, Karlsruhe, Germany) with pH 6 was used to culture *L. monocytogenes* DSM 20600. Plate count agar (PCA) (Carl Roth GmbH & Co. KG, Karlsruhe, Germany) was used to make 92 mm plates for dilution plating and colony counting for all bacterial species during the growth curves. Two overnight cultures of each species were made by adding 1 colony to 20 mL of the respective broth (TSB or BHI) and were statically incubated for 19 h prior to starting the sample cultures for the growth curves. *E. coli* and *S. aureus* were incubated at 37 °C while *B. cereus* and *L. monocytogenes* were incubated at 30 °C. The optical density at 600 nm (OD_600_) of each overnight culture was measured with 1 mL of a 1:5 dilution (UV-3100PC spectrophotometer from VWR International GmbH, Darmstadt, Germany) using 1 mL of the respective starting broths as the blank. The overnight cultures were then adjusted to create a 200 mL culture with an OD_600_ of 0.02.

Other solutions prepared for the experiment included 0.85% NaCl (Carl Roth GmbH & Co. KG, Karlsruhe, Germany) for sample preparation and 0.85% NaCl with 0.1% tryptone (AMRESCO, VWR International GmbH, Darmstadt, Germany) to perform serial dilutions.

### 2.3. PTW Treatment of Sample Culture and Growth

For each individual bacteria species, 5 mL of the overnight culture was centrifuged (Eppendorf centrifuge 5910R/Ri with rotor S-4x universal, Eppendorf SE, Hamburg, Germany) for 5 min at 4500 rpm and room temperature (RT) to form a pellet. The supernatant was then removed and the pellet was dissolved in 5 mL of 0.85% NaCl solution. This suspension of bacteria was then adjusted to create a final volume of 200 mL with 0.85% NaCl in a 1 L Eppendorf centrifugation bottle (Eppendorf article no. 5910770.006) with an OD_600_ = 0.02, using 0.85% NaCl solution as the blank. To the 200 mL of adjusted bacteria, 28.6 mL of PTW was added and incubated for 5 min at (RT) while shaking at 75 rpm. Subsequently, PTW-bacteria suspension was stopped by adding an equal amount of the respective TSB or BHI broth (228.6 mL), and followed by centrifugation for 5 min at 4500 rpm. Then, the supernatant was removed and the treated pellet was resuspended in 200 mL TSB or BHI broth in the centrifugation bottle before finally being transferred into a 600 mL glass flask. The control samples were treated without the centrifugation step; instead 5 mL of the overnight culture was adjusted to OD 0.02 in 200 mL broth. This occurred at time point 0 in the control growth curves. Both the control and PTW-treated samples for each bacteria species were allowed to grow as shown in Table 1. Based on preliminary tests with untreated but centrifuged controls, no strong influence of the extra centrifugation on the obtained cell numbers was detected. The length of growth time depended on when the bacteria colonies no longer increased in number, indicating that it had reached the stationary phase, based on optical density at 600 nm and not on cell count.

### 2.4. OD Measurement and Colony-Forming Unit (CFU) Detection

To determine the OD at different time points during bacterial growth, three 1 mL samples were taken from the bacterial suspension under aseptic conditions, transferred into separate cuvettes and independently photometrically measured at OD_600_. In total, 3 biological replicates at each time point were measured, and each experiment was repeated at a minimum of 3 times to gain 3 experimental replicates. The minimum number of replicates was n = 9.

To determine the CFU/mL, another 1 mL of the bacterial suspension at each specific time point was transferred into a 1.5 mL Eppendorf tube and used for serial decimal dilutions. Subsequently, 10 μL of each dilution was pipetted onto agar and spread by tilting the plate up and down. The grown colonies were counted manually after 24 h of incubation at the optical temperatures given in Table 1. The limit of detection is defined as the smallest detectable amount of the target organism in the sample under investigation. In the lowest dilution (10 µL), it was assumed that a single colony could be detected. Therefore, the limit of detection was 0.01 (≥100 CFU/mL). Each sample was analyzed with 3 biological replicates and a minimum of 3 experimental replicates, resulting in n = 9 replicates.

The time points of detection for OD_600_, hence also for CFU, were every hour from 0 h to 9 h (control). The same points for the treated *E. coli* samples were used, with additional measurements at 5.5 h, 6.5 h, 7.5 h, 10 h, and 11 h. For *B. cereus*, measurements were taken every hour from 0 to 12 h (control). For the treated samples, measurements were taken at 0 h and then hourly from 12 h to 23 h. In the case of *S. aureus*, the measurement time points were hourly from 0 to 11 h (control), and 0 h, 4 h, 6 h, and hourly from 8 h to 22 h for the treated samples. Finally, the measurement time points were hourly from 0 to 15 h (control), and 0 h, 4 h, 6 h, and hourly from 8 h to 24 h for *L. monocytogenes*.

CFU/mL was calculated as follows:

$$CFU=\frac{10^{x}}{V}\times \frac{\sum c_{x}+\sum c_{x+1}}{n_{x}+{0.1n}_{x+1}}$$

10*^x^*: dilution factor for the lowest dilution;
V: volume of diluted cell suspension in ml;
n_x_: number of plates used for the lowest evaluated dilution;
n_x+1_: number of plates used for the next highest evaluated dilution;
∑ c_x_: total number of CFU counted on n_x_;
∑ c_x+1_: total number of CFU counted on n_x+1_.

### 2.5. Growth Data Analysis

The individual descriptive growth coefficients were calculated from the growth data (CFU and OD) using a logistic growth model. First, the logistic growth model according to Levins (1969) was defined (Equation (1)) [35]:

(1) $$\frac{dN}{dt}=\mu \cdot N\cdot \left(1-\frac{N}{K}\right)$$

with the analytical form:

$$N\left(t\right)=\frac{K}{1+\left(\frac{K-N_{0}}{N_{0}}\right)\;e^{-\mu t}}$$

where:

*N*(*t*): cell count (either as CFU or OD as surrogates) at time *t*;
*μ*: specific growth rate (h^−1^);
*K*: carry capacity.

The lag phase was then geometrically determined from the logistic model using the tangent method [36,37,38]. The intersection of one tangent in the initial cell count/ initial OD N_0_ and the second tangent in the inflection point N_infl_ of the logistic growth curve marks the lag phase duration. The inflection point was determined by setting the derivative of (Equation (1)) to 0 (Equation (2)).

(2) $$\frac{d^{2}N}{dt^{2}}=\;\mu^{2}\cdot N\cdot \;\left(1-\frac{N}{K}\right)\cdot \;\left(1-\frac{2N}{K}\right)=0$$

Equation (2) is equal to 0 for:

$$\begin{array}{c} (i)\;\;N=0\;for\;\;\;K\neq 0\\ (ii)\;\;\;N=K\;for\;\;\;K\neq 0\\ \left(iii\right)\;\;\;N=\frac{K}{2}\;for\;\;\;K\neq 0\\ \left(iv\right)\;\;\;\mu =0 \end{array}$$

Solutions (i) and (ii) are the starting and end conditions of the bacterial growth. Solution (iv) implies no bacterial growth, which results in a constant cell count $N\left(t\right)=N_{o}\;\forall \;t\;in\;[0,\;\infty ),$ and thus no inflection point. Therefore, solution (iii) was selected as the inflection point for the tangent method.

$$N_{infl}=\frac{K}{2}\;for\;K\neq 0$$

## 3. Results

### 3.1. Growth Curves Based on Optical Density (OD)

**Figure 1:**
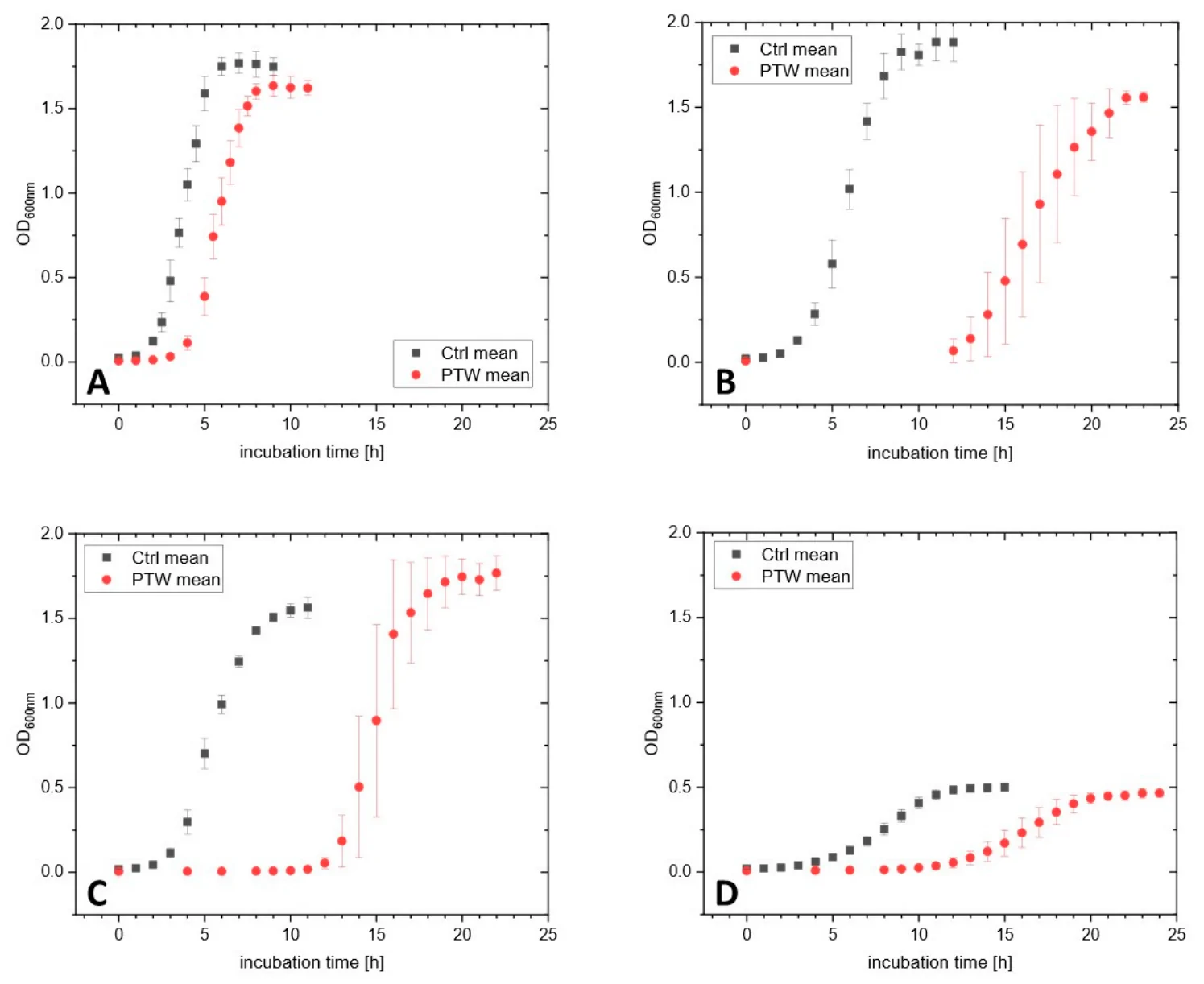
Growth curves of *E. coli* (**A**), *B. cereus* (**B**), *S. aureus* (**C**), and *L. monocytogenes* (**D**) without (black) and with (red) PTW treatment based on optical density at 600 nm after incubation. Error bars show the standard deviation of the average OD values based on minimum n = 9 datapoints.

The results of the growth curves based on the OD at 600 nm are given in Figure 1. In each graph, the OD over incubation time is given for the untreated and PTW-treated samples. Depending on the bacteria, the incubation time for the control samples varied, which was expected. However, the PTW treatment influenced the Gram-positive bacteria particularly strongly by effecting the length of the lag phase. Apart from *L. monocytogenes*, each of the bacteria reached an OD between 1.5 and 2.0 in the early stationary phase. *L. monocytogenes* reached an OD of 0.5 in the same time, which may be explained by the smaller individual size of the bacterium. The size of *E. coli* is 1.1 to 1.5 µm in diameter and 2.0 to 6.0 µm in length [39]. Comparable have been reported for *Bacillus* species, which range from 0.5 to 1.2 µm in diameter and 2.5 to 10 µm in length [40]. *L. monocytogenes* is comparably small, ranging from 0.4 to 0.5 µm in diameter and 0.5 to 2.0 µm in length [41]. The size of the coccus *S. aureus* ranges from 0.5 to 1.5 µm [42].

**Figure 2:**
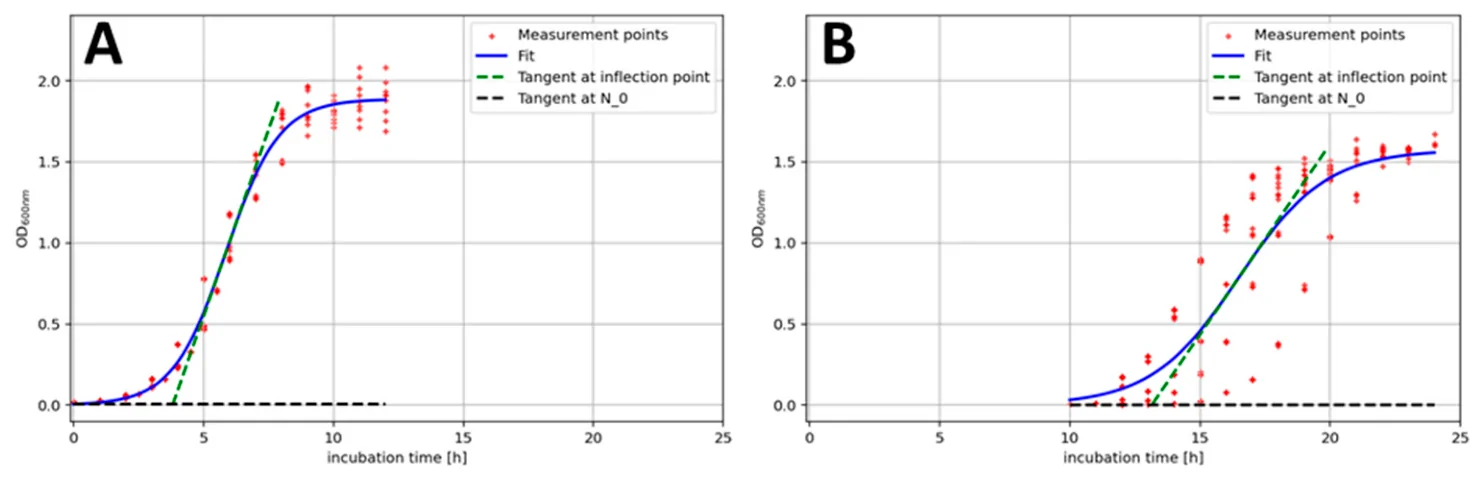
Fit of the logistic growth model and the applied tangents for the two-tangent method for the control (**A**) and treatment (**B**) of *B. cereus* based on optical density at 600 nm after incubation.

The description of bacterial growth by the logistic model based on optical densities is illustrated in Figure 2, using *B. cereus* as an example.

### 3.2. Growth Curves Based on Colony-Forming Units (CFUs)

**Figure 3:**
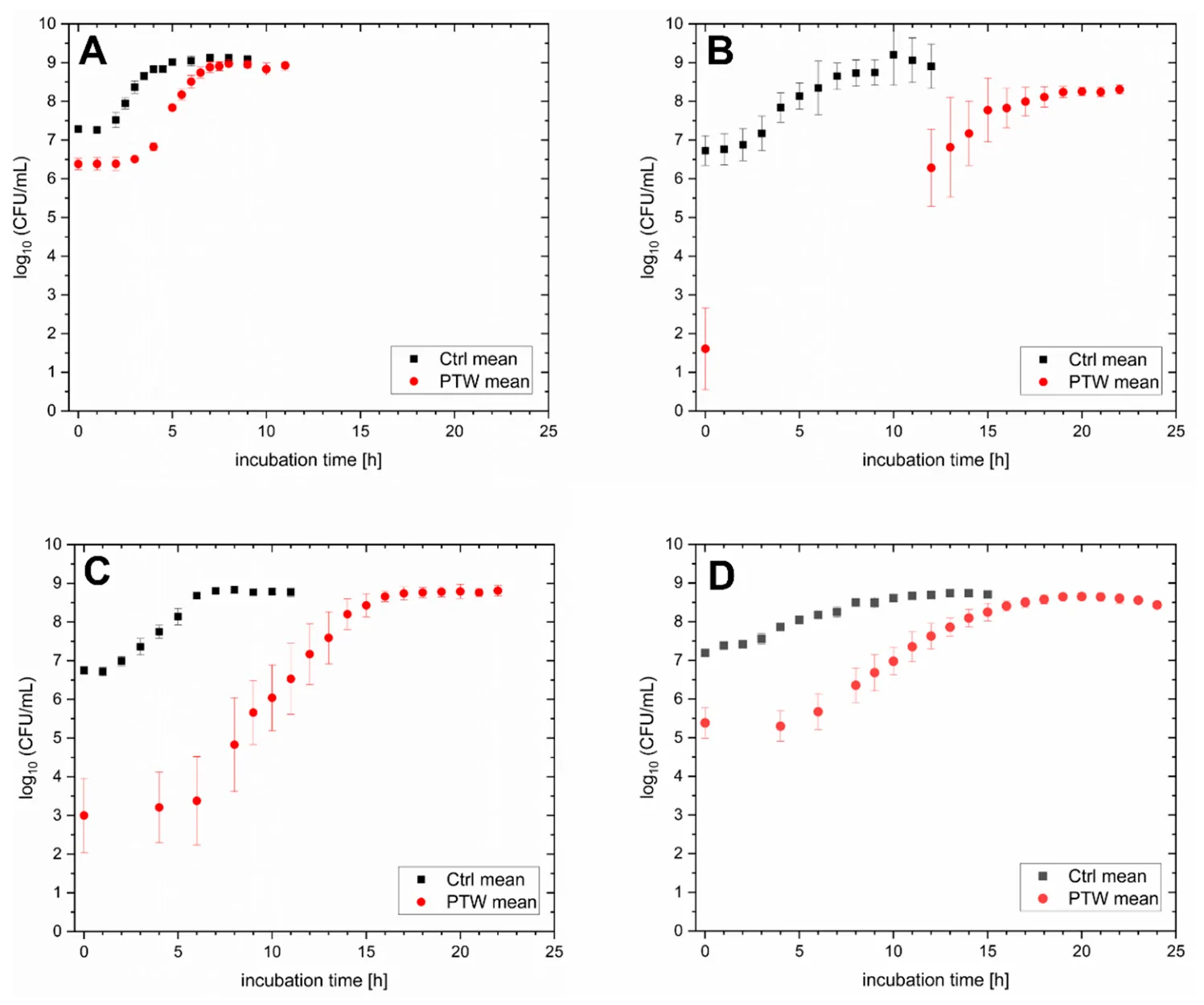
Growth curves of *E. coli* (**A**), *B. cereus* (**B**), *S. aureus* (**C**), and *L. monocytogenes* (**D**) without (black) and with (red) PTW treatment based on manually counted colony-forming units (CFUs) per mL after incubation. Error bars show the standard deviation of the average logarithmic values of CFU/mL based on minimum n = 9 datapoints.

The growth curves of the colony-forming units of each bacterium without and with PTW treatment (Figure 3) support the hypothesis of lag phase elongation by PTW treatment. The curve pattern differs depending on the individual bacterium; however, the CFU/mL values at time point zero for the control samples are all close to 7 log_10_ cycles. The CFU/mL values for the PTW-treated samples decreased moderately (in the case of *E. coli* and *L. monocytogenes*) or dramatically (in the case of *B. cereus and S. aureus*). With a delay of 2 h for *E. coli* up to 11 h for *B. cereus*, the CFU/mL numbers reached stationary phase values comparable to those of the non-treated ones. The detected differences in lag phase length and slope of the exponential phase between the non-treated and PTW-treated curves may be caused by cell death, induction viable-but-nonculturable (VBNC) state and the resulting reduced metabolism, or, in the case of *B. cereus*, endospore formation triggered by oxidative stress and the resulting increased metabolism. Further analyses and investigations would be needed to differentiate the observed effects in detail.

**Figure 4:**
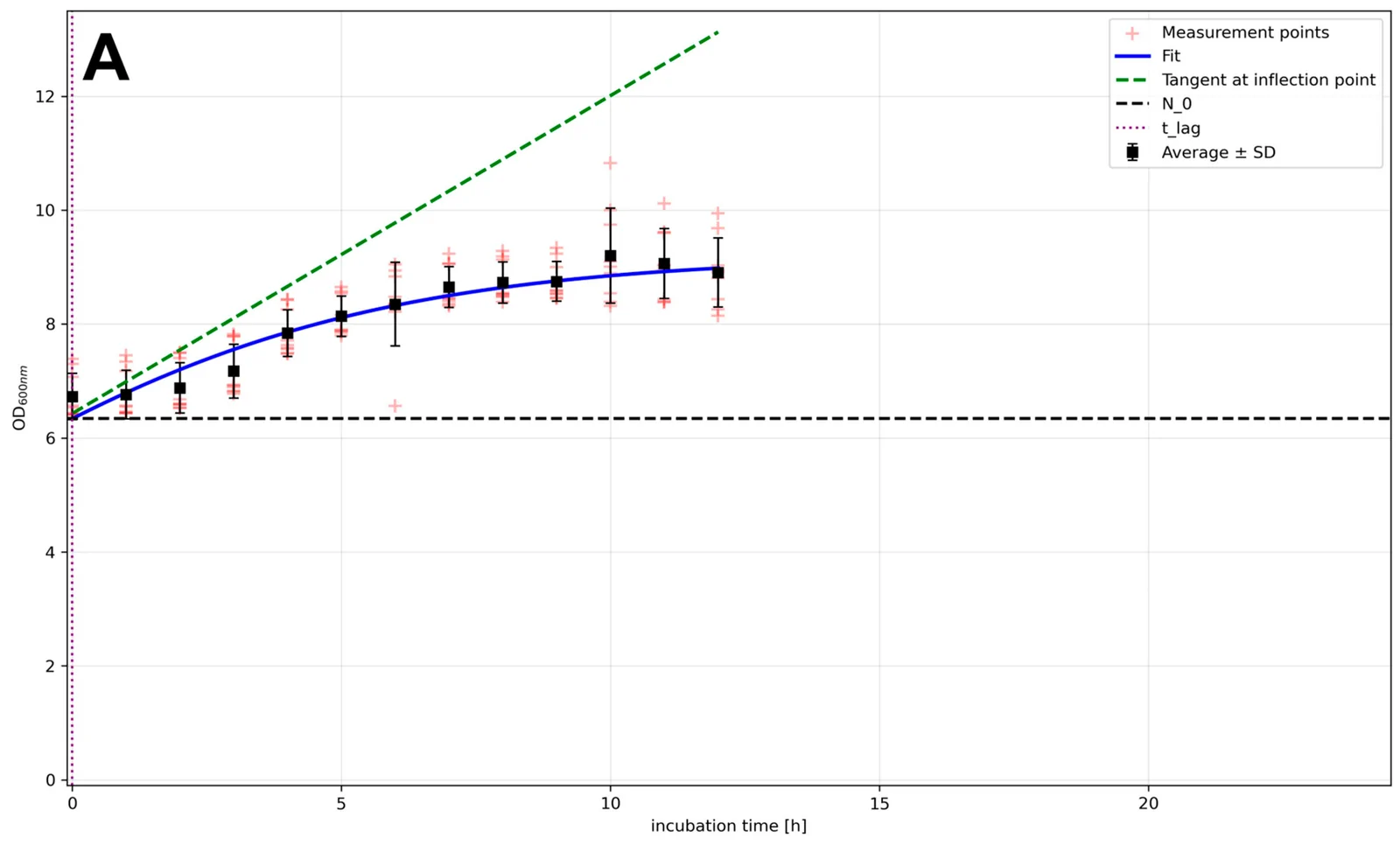

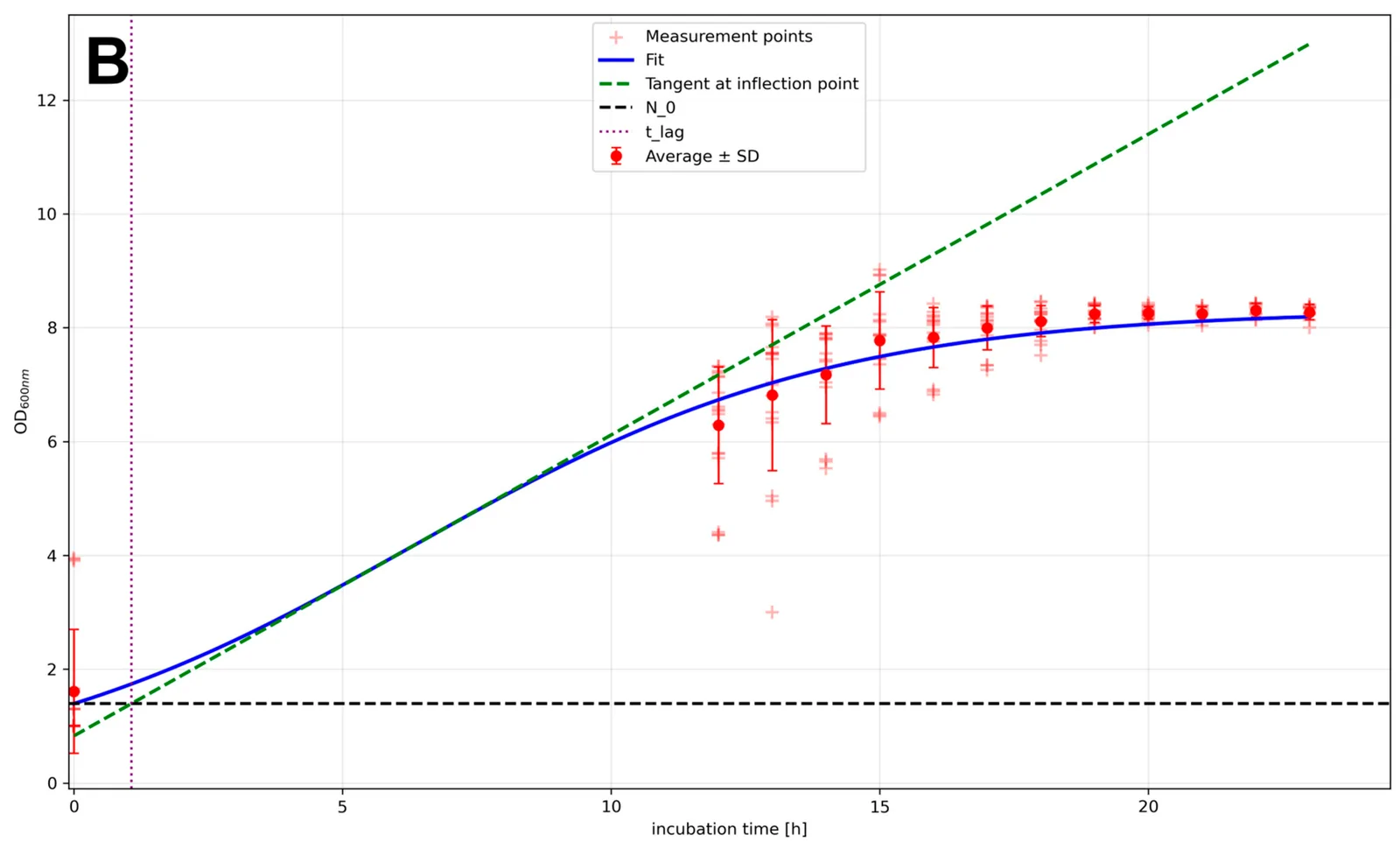
Fit of the logistic growth model and the applied tangents for the two-tangent method for the average CFU/mL for control (**A**) and treated (**B**) *B. cereus*-based colony-forming units after incubation. The error bars show the standard deviation.

Similarly, to the OD data, the growth curves based on the CFU data are described using the logistic model and illustrated using *B. cereus* as an example in Figure 4.

### 3.3. Comparison of the Growth Model Parameters for OD and CFU Data

The individual model parameters of the logistic models fitted to each microorganism are summarized in Table 2.

## 4. Discussion

The results of the growth curves show a strong effect of PTW treatment on the lag phase of bacterial proliferation, depending on the specific bacteria. Here, the difference between Gram-negative bacteria (Figure 1A and Figure 3A) and Gram-positive bacteria (Figure 1B–D and Figure 2) was greater than between different Gram-positive bacteria (e.g., Figure 1B–D). In general, the OD-based growth curves of the control samples are comparable to those found in the literature [43,44,45,46]. This could mean that the differences in growth of the PTW-treated samples are attributable to the PTW treatment. The difference in the handling of the treatment and control samples has to be acknowledged as a potential methodological limitation. However, reasons for this observed difference, especially in the lag phase, can be a combination of cell death induced by PTW’s antibacterial properties and/or the VBNC state induced by oxidative stress due to PTW’s reactive species [47]. The antibacterial properties of PTW are well known [48,49] and attributed to its low pH, high conductivity, strong Oxidation Reduction Potential (ORP), and the presence of reactive oxygen and nitrogen species. The presence of RONS in PTW may also induce the so-called VBNC state, with drastic reduced metabolic activity in the bacteria [50]. In the case of *B. cereus*, the oxidative stress may trigger spore formation as a survival strategy [51]. A general overview of the possible mechanisms of action of hydrogen peroxide and reactive nitrogen species in PTW against Gram-positive and Gram-negative bacteria was given by Schnabel et al. (2021) [52]. PTW is an acidic water enriched with RONS. These conditions can damage bacteria, so bacteria may respond by activating stress defense systems against the following: (1) acidic stress due to the very low pH (<2), which can induce proton pumping, increase intracellular pH, or activate acid-resistance genes; (2) oxidative stress due to ROS in PTW, which can lead to the production of catalases, superoxide dismutase, and peroxidases as ROS, possibly damaging bacterial membranes, proteins, and DNA; (3) nitrosative stress due to RNS in PTW, which can activate detoxification and repair systems to avoid interference with metabolism, preventing damage to cellular components; (4) general envelope stress, as PTW components could disrupt the cell membrane and cell wall, resulting in changes in membrane composition or the activation of protective membrane stress pathways; (5) DNA/protein repair response. These stress responses may make bacteria temporarily more tolerant to PTW treatment, but if PTW exposure is strong enough, the damage may exceed their defense capacity and lead to inactivation.

When considering the physical properties of bacterial OD measurements, the Mie scattering theory and the Rayleigh–Gans approximation for optically “soft” particles provide the general framework. The Rayleigh–Gans approximation accounts for the small difference in refractive index between bacterial cells (n ≈ 1.38) and the surrounding medium (e.g., water n ≈ 1.33). At a wavelength of 600 nm, forward light scattering separates particles with sizes of 0.5 µm and larger from lower particles [53,54]. As the chosen bacteria in our investigations are 0.5 µm or larger in their alive state, only cells with intake envelopes would lead to light scattering independent from their metabolic state or DNA effects. Therefore, the low OD values for PTW-treated bacteria at time point zero should be a result of damaged cell envelopes and dead cells. The low number of envelope/shape intact cells may contribute to the lag phase elongation, as time is needed to recover from stress and to reach cell numbers that trigger the exponential phase. However, the data presented here provide very basic insights into the influence of PTW on the growth behavior of different bacteria. Due to the experimental and modeling setup, a clear distinction between the reduction of cell number and/or dormancy of cells due to oxidative stresses is not possible. Therefore, further analyses are needed to investigate these mechanisms in more detail, especially regarding metabolic activity, the production of internal and external metabolites, and cell envelope and DNA structure.

In the context of PTW application within food processing chains, it is necessary to understand if the observed influence on the lag phase is also achieved on real food. Here, the research question on transient versus permanent stress on the bacteria is important, as permanent stress may lead to cell death, whereas transient stress may lead to the VBNC state, spore formation or just a delay in specific growth phases. However, both types of stresses have advantages. Cell death leads to a lower microbial load on the food matrix and lowers the foodborne illness risk. A delay in growth or VBNC stress may provide more time for avoiding attachment (e.g., biofilm growth), as sanitizers may be used while bacterial numbers are still low. If spore formation is induced, the challenge of sanitation would increase as bacterial spores are very hard to inactivate.

As the PTW treatment was applied to cells in suspension at a stage corresponding to the initial step of biofilm formation on surfaces, when low numbers of bacteria in the environment start to attach and subsequently proliferate, it would be interesting to investigate the behavior of the investigated strains under biofilm growth conditions (i.e., on a solid surface rather than in suspension) and compare the growth curves. A comparable outcome under these conditions would mean that PTW is able to reduce or slow the biofilm formation, which would be beneficial for the food processing industry. The ability of PTW treatment to efficiently reduce existing biofilms has already been demonstrated [30,31,55]. Handorf et al. (2020) showed strong antimicrobial effects of PTW against *Pseudomonas fluorescens* biofilms. PTW treated with microwave-induced plasma for 300 s and applied to *P. fluorescens* biofilms for 5 min reduced colony-forming units by up to 6 log_10_. The strongest treatment (900 s pretreatment, 5 min exposure) reduced cell viability by 81% and metabolic activity by 92%. Microscopy confirmed biofilm inactivation even under the shortest treatment conditions, along with reduced biofilm thickness and increased cell clustering. These effects were associated with a lower pH of the plasma-treated water [30]. For *Listeria monocytogenes* biofilms, Handorf et al. (2021) [55] also achieved pronounced reductions with PTW. The CFU decreased by 4.7 log_10_, metabolic activity by 47%, and cell vitality by 69% compared with controls. LIVE/DEAD staining and fluorescence microscopy showed reduced viability with longer treatment and incubation times, and AFM revealed structural changes in the biofilm [55]. Nestel et al. (2025) [31] also showed that PTW effectively reduced the proliferative ability of Gram-positive bacterial biofilms in food processing environments. However, its effects differed between species: *Listeria monocytogenes* showed less membrane damage but a stronger decrease in metabolic activity, suggesting possible induction of a VBNC state, while *Bacillus cereus* appeared to respond by sporulation. These findings indicate that PTW is an effective sanitation method, though bacterial survival strategies may vary [31].

The data presented in Table 2 show the model parameters of the fitted logistic model (Equation (1)) on the measurement data. The model fit to the OD data (illustrated in Figure 2) provides a good representation of the real measurement data and a good geometric estimation of the lag duration by the shown tangents. In the case of the CFU data, the averages of each timepoint were taken into account, as the standard deviation distorts the fitting. To remedy some of the distortion, the carry capacity K was fixed for the CFU fits. The fits for the CFU data are of poor quality, as the alignment with the real measurement data is affected by the standard deviations. Therefore, the geometric estimation of the lag duration indicates the passing of the lag phase before the first sampling for most microorganisms (except *B. cereus* and *S. aureus* treatment). This event relates to the time offset from sampling from the cultivation to the CFU plating. In general, better agreement between the model and the measured data can be observed for the OD data. The CFU data, which are more susceptible to noise, should be analyzed using a more biological adapted model (such as the Baranyi-Roberts [56] or Gompertz [57] model), which allows more domain-specific descriptions by more biologically related parameters. Therefore, the interpretation of the model data for CFU curves includes more bias. Furthermore, it would be useful to expand the model by including parameters relevant to plasma treatment in order to account for the treatment effects on the growth dynamics of the microorganisms. The amount of data also needs to be increased to better represent real-world growth processes, since the amount and quality of the data are crucial for improving model precision.

Published studies have shown that PTW can inactivate a broad range of bacteria, including major foodborne pathogens such as *E. coli*, *L. monocytogenes*, *S. aureus*, and *B. cereus* [58]. The antimicrobial effect is generally attributed to reactive oxygen and nitrogen species, especially hydrogen peroxide, nitrite, nitrate, and related acidic conditions. These compounds can damage cell membranes, proteins, DNA, and metabolism [52].

The present work is novel in its use of a microwave-driven discharge with compressed air to produce well-characterized plasma-processed air [32], which was then used to treat tap water and produce a PTW with defined chemical properties [31]. Compared with many previous PTW studies that have relied on plasma jets, dielectric barrier discharges, or synthetic gases, this approach is based on inexpensive air and water and therefore has higher practical potential. A second novelty is the investigation of four foodborne pathogens with distinct resistance traits and their growth behavior over time after treatment. Previous studies have often focused on single strains, biofilms, or short fixed timepoints. In contrast, this work examined recovery and growth over a longer period, providing new insight into survival dynamics and possible early attachment processes relevant to biofilm development.

Overall, the study combined well-characterized plasma chemistry, multiple important foodborne pathogens, and growth-phase analyses, offering both novelty and practical significance for food safety applications.

## 5. Conclusions

Plasma-treated water (PTW) clearly prolonged the lag phase of the investigated foodborne pathogens, with a more pronounced effect on Gram-positive species than on *E. coli*. Logistic growth modeling of optical density and colony-forming unit data showed a trend of delayed growth kinetics after treatment and indicated species-specific differences in response. However, the risk of noise-related distortions in the model application on the CFU data has to be kept in mind. Although PTW reduced initial cultivability, most bacteria ultimately recovered and reached the stationary phase after a delayed growth period. These findings suggest that PTW induces transient antimicrobial stress rather than complete inactivation under the conditions tested, potentially involving membrane damage, VBNC induction, or sporulation in *B. cereus*. Further studies are needed to validate these findings in real food matrices and biofilm systems and to refine statistical models for PTW-treated microorganisms.

## Figures and Tables

**Table 1:** Investigated bacteria, their optimal growth temperature for incubation and growth time for control and treated samples.

Bacteria	Temperature (°C)	Length of Growth (h)—Control Sample	Length of Growth (h)—PTW Sample
*B. cereus*	30	12	23
*L. monocytogenes*	30	15	24
*E. coli*	37	9	11
*S. aureus*	37	11	22

**Table 2:** Average growth curve parameters with their standard deviations (SD) from the fitted logistic model for the tested microorganisms (* = fixed parameter; ** = fitting inflection point already passed before first sampling).

Logistic Model Parameters Based on Optical Density Data									
Species	Dataset	µ [1/h]	SD	K	SD	N_0_	SD	t_inf_ [h]	t_lag_ [h]
*E. coli*	control	1.4841	±0.0515	1.7717	±0.0116	0.007128	±0.001347	3.71	2.37
	treatment	1.4019	±0.0563	1.6385	±0.0147	0.000515	±0.000166	5.75	4.33
*B. cereus*	control	0.9744	±0.0366	1.8854	±0.0175	0.006217	±0.001268	5.86	3.81
	treatment	0.5975	±0.0662	1.5733	±0.0647	0.000083	±0.000086	16.49	13.15
*S. aureus*	control	0.9522	±0.0199	1.5569	±0.0071	0.009491	±0.000986	5.35	3.25
	treatment	0.8255	±0.0756	1.7483	±0.0360	0.000011	±0.000012	14.47	12.05
*L. monocytogenes*	control	0.5438	±0.0177	0.5231	±0.0062	0.007067	±0.000874	7.89	4.21
	treatment	0.5151	±0.0302	0.4819	±0.0108	0.000122	±0.000056	16.08	12.20
Logistic model parameters based on colony-forming unit data									
Species	dataset	µ [1/h]	SD	K *	SD *	N_0_	SD	t_inf_ [h]	t_lag_ [h]
*E. coli*	control	0.4202	±0.0627	9.1278	/	6.9018	±0.2170	0 **	0 **
	treatment	0.3878	±0.0522	8.9766	/	5.6820	±0.3510	0 **	0 **
*B. cereus*	control	0.2426	±0.0244	9.2020	/	6.3390	±0.1631	0 **	0 **
	treatment	0.2545	±0.0160	8.3079	/	1.3943	±0.2322	6.29	1.07
*S. aureus*	control	0.3164	±0.0469	8.8347	/	6.3415	±0.2123	0 **	0 **
	treatment	0.2577	±0.0266	8.8143	/	1.4277	±0.2952	6.38	1.13
*L. monocytogenes*	control	0.2221	±0.0175	8.7354	/	7.0267	±0.0811	0 **	0 **
	treatment	0.1598	±0.0171	8.6497	/	4.4176	±0.3150	0 **	0 **

## Data Availability

The original contributions presented in this study are included in the article. Further inquiries can be directed to the corresponding author.
